# The relation between intraocular pressure change and plasma natriuretic peptide under simulated hypobaric conditions

**DOI:** 10.4103/0301-4738.62642

**Published:** 2010

**Authors:** Remzi Karadag, Ahmet Sen, Nilgun Yildirim, Hikmet Basmak, Haydar Golemez, Erdinc Cakir, Ahmet Akin

**Affiliations:** Department of Ophthalmology, Eskisehir Military Hospital, Eskisehir, Turkey; 1 Department of Aerospace Medicine, Gulhane Military Medical Academy, Eskisehir, Turkey; 2 Department of Ophthalmology, Eskisehir Osmangazi University Hospital, Eskisehir, Turkey; 3 Department of Biochemistry, Gulhane Military Medical Academy, Ankara, Turkey

**Keywords:** Brain natriuretic peptide, high-altitude, hypobaric chamber, hypoxia, intraocular pressure

## Abstract

**Purpose:**

To ascertain whether the changes in intraocular pressure (IOP) that occur during hypobaric hypoxic exposure are related to plasma N-terminal pro-brain natriuretic peptide (BNP) levels.

**Materials and Methods:**

The study group comprised 26 healthy participants (all male, mean age 23.1 years). IOP was measured at local ground level, (792 m above sea level), then while in a chamber providing hypobaric hypoxic conditions (the subjects were exposed to a pressure equivalent to 9144 m for 1-3 min), and again after exit from the chamber. In each condition, the mean of three consecutive measurements of IOP was calculated for each eye. For BNP measurements, blood samples were drawn before the participants entered the chamber and just after they left the chamber.

**Results:**

IOP during hypobaric hypoxic exposure (18.00 ± 3.70 mmHg) was significantly greater than that before (15.66 ± 2.10 mmHg, *P* < 0.001) or after (16.10 ± 2.63 mmHg, *P* = 0.001) the exposure. IOP levels before and after the exposure were not significantly different (*P* = 0.136). Plasma BNP levels measured before and after exposure to hypobaric hypoxic conditions were not significantly different (*P* = 0.462).

**Conclusion:**

Plasma BNP levels did not change after short-term hypobaric hypoxic exposure, while the IOP increased. This increase may have been caused by some other systemic factors. As the hypobaric hypoxic conditions were reversed, IOP decreased to normal levels.

High altitude has diverse systemic and ophthalmic effects on individuals. Ophthalmic effects include changes in the conjunctiva, cornea, lens, retina, optic nerve, and intraocular pressure (IOP).[1,2]

IOP at high altitude has been a topic of controversy for many years. Some studies have found IOP to be reduced under natural[3,4] or simulated[5] high altitude conditions, while others have found it to be raised[6–8] or normal.[1,9] The increased level of IOP at simulated hypobaric hypoxic conditions returns to nearly previous level when the subject descends to ground level.[6]

The mechanism of IOP changes that occur under high altitude conditions remains unclear.[4,9] It is known that IOP measurements can be affected by changes in central corneal thickness (CCT).[10,11] Furthermore, recent studies found that hypobaric hypoxic exposure can cause an increase in CCT,[12,13] however, in our study this CCT increase was not enough to explain the change in IOP.[14]

It has been shown that brain natriuretic peptide (BNP) can reduce IOP.[15,16] In rabbits, intravitreally injected BNP has been found to increase cGMP concentration in the aqueous humor, resulting in an increase in outflow.[17] In porcine eyes, BNP-like immunoreactivity-containing nerve fibers are found in the aqueous humor outflow pathway, ciliary processes, and anterior ciliary muscles.[18] In rat eyes, there is also expression of BNP mRNA in the retina, choroid, and ciliary body.[19] These studies suggest that BNP may have a role in the regulation of IOP.

Plasma levels of BNP have been found to increase during hypoxia.[20] In addition to extended exposure to hypobaric hypoxia itself (10-91 days), endurance training in hypobaric hypoxic conditions leads to a marked early increase in ventricular and atrial BNP mRNA levels.[21] As for the physiologic effects that can be produced by elevated plasma BNP levels, Klinger *et al*., found that in rats, BNP infusion attenuated the development of hypoxic pulmonary hypertension.[22] The authors suggested that this finding supports the hypothesis that endogenous BNP plays a role in modulating the pulmonary hypertensive responses seen in chronic hypoxia. Since we found in our previous study that the changes in CCT in hypobaric hypoxic conditions have minor effect on the changes in IOP,[14] we planned the current study by reapplying some data of the previous study to evaluate the relationship between IOP and plasma BNP levels. To the best of our knowledge, such an association has not been explored in the past. We hypothesized that the changes in IOP which occur under hypobaric hypoxic conditions might be associated with plasma BNP levels. We hypothesized that the change in IOP under hypobaric hypoxic conditions, which has been shown previously, may be compensated and returned to normal values with the increasing BNP under these conditions. We aimed to examine whether the increased IOP caused by hypobaric hypoxic exposure is compensated by plasma BNP level changes.

## Materials and Methods

The study group comprised 26 male pilots (52 eyes) with a mean age of 23.1 ± 1.6 years (range, 21-34 years). The study was conducted in accordance with the Helsinki Declaration and was approved by the local institutional ethics committee. For each participant, a full ophthalmic examination, including refraction, a slit-lamp examination, gonioscopy, and assessment of the posterior segment was carried out. None of the subjects had a history of ocular disease.

Measurements of IOP were made in both eyes in each participant under topical anesthesia with proparacaine hydrochloride. A Tono-Pen XL tonometer (Medtronic-Solan, Jacksonville, USA) was utilized to measure IOP. The mean of three consecutive measurements of IOP was recorded for each eye. The Tono-Pen XL® has been reported to be unresponsive to alterations in ambient barometric pressure.[1,5,6]

An altitude chamber (ETC, Philadelphia, USA) with a capacity of 10 trainees and two inside observers was used for hypobaric hypoxic simulation.

**Figure 1:**
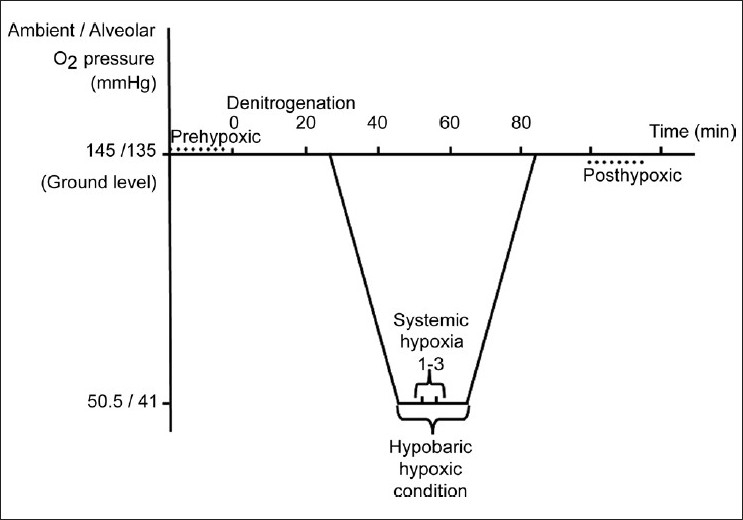
The timeline of hypobaric chamber flight

IOP was measured at local ground level, which is 792 m (2598 ft) above sea level, 10 min before participants entered the hypobaric chamber (prehypoxic condition). At this altitude the partial pressure of oxygen (pO_2_) is 145 mmHg and alveolar pO_2_ is 135 mmHg. Denitrogenation was achieved by breathing 100% O_2_ with a tight-fitting pilot mask for 30 min. This mask covered just the mouth and nose, not the eyes. In the chamber, the temperature was approximately 24°C and relative humidity was 32% at ground level conditions. Then the chamber was decompressed to a simulated altitude of 30000 ft (9144 meters) in 20 min, which decreased the pO_2_ to 50.5 mmHg and alveolar pO_2_ to 41 mmHg. The temperature decreased to 22°C at the simulated altitude. This altitude was chosen because 30000 ft is the regular flight altitude for most airline transport aircraft. Aviators or passengers may get exposed to this level of low pressure due to a cabin pressure loss or an increase in altitude without cabin pressurization. At the simulated target altitude, participants removed their oxygen masks and breathed ambient air for 1-3 min. When their performance of the pen-paper (orientation) test was observed to be impaired (hypobaric hypoxia), the mask was donned immediately and IOP measurements were performed. At a simulated altitude of 30000 ft with 100% oxygen, arterial oxygen saturation is maintained at 97%, but it immediately goes down to 66% or below–even at lower altitudes–where subjects show the signs of cognitive impairment.[23] IOP measurements were repeated at ground level about 10 min after participants left the chamber (posthypoxic condition). All IOP was measured in the sitting position with the same instrument for all participants and instrument was calibrated before each session. The timeline of the procedures in the hypobaric chamber is illustrated in Fig. 1.

For plasma BNP measurements, blood samples were drawn 30 min before the participants entered the chamber, and immediately after they left the chamber. BNP levels during hypobaric hypoxic conditions were not measured due to the difficulty in drawing blood during hypoxia training. Because of the long plasma half-life of N-terminal pro-brain natriuretic peptide[24] we considered that the value after the chamber session also reflected the hypoxic value. Plasma BNP levels were measured with an Elecsys 1010 (Roche, Switzerland).

All statistical analyses were performed with the use of SPSS for Windows, Version 13.0 (Chicago, USA). Unless otherwise stated, results were expressed as mean ± standard deviation. *P* values of less than 0.05 were considered statistically significant. Repeated measures ANOVA and the Wilcoxon test (two related samples tests) were used as appropriate.

## Results

All subjects had normal ophthalmologic findings and best-corrected visual acuity was 20/20 or better in each eye.

In the 52 eyes of the 26 participants the mean prehypoxic IOP was 15.66 ± 2.10 mmHg (range, 10-22 mmHg); hypobaric hypoxic IOP was 18.00 ± 3.70 mmHg (range, 11-26 mmHg), and posthypoxic IOP was 16.1 ± 2.63 mmHg (range, 10-23 mmHg). IOP under hypobaric hypoxic conditions was significantly greater than both prehypoxic (*P* < 0.001) and posthypoxic (*P* = 0.001) IOP. There was no significant difference between pre- and posthypoxic IOP levels (*P* = 0.136).

Before the participants entered the chamber for hypobaric hypoxic exposure, their mean plasma BNP level was 26.8 ± 15.7 pg/ml (range 6.2-67.9 pg/ml). Immediately after exit from the chamber, the mean plasma BNP level was 24.2 ± 17.4 pg/ml (range 6.1-81.3 pg/ml). These pre- and post-exposure BNP levels did not differ significantly from each other (*P* = 0.461).

## Discussion

The research literature regarding the effects of hypobaric hypoxic exposure on IOP has not yet provided a clear picture of the mechanisms involved. Another complicating factor is that CCT, which can change in response to hypobaric hypoxic conditions,[12,13] can thereby lead to artifactually high measurements of IOP. In our previous study we have found that the change in CCT was not enough to explain the increase in IOP in hypobaric hypoxic conditions.[14] The reason why the IOP returns to the pre-exposure values at the end of the hypobaric exposure is not clear, either. The rationale for this study was therefore to assess the validity of BNP as a possible factor in the regulation of IOP under hypobaric hypoxic conditions.

This study is consistent with some previous studies[6–8] which demonstrated that hypobaric hypoxia results in a significant increase in IOP. Some studies concluded that IOP measurements were affected by high altitude environmental conditions. Ortiz *et al*.[25] reported that cold air led to a drop in IOP due to a fall in episcleral venous pressure. Passo *et al*.[26] showed that exercise and fatigue caused a fall in IOP. Since our study has been conducted in a hypobaric chamber, the effect of cold air, fatigue and exercise was not present.

Natriuretic peptides are known to have effects on IOP.[16] In a study by Takashima *et al*.,[17] BNP was found to induce a significant reduction in IOP when injected intravitreally into the rabbit eye. The injected BNP led to an increase in cGMP concentration in the aqueous humor, and to increased aqueous outflow. Fernández-Durango showed that intracamerally injected BNP stimulated guanylate cyclase activity and decreased IOP.[16] Under chronic hypoxic conditions, plasma levels of BNP have been found to increase.[20] In addition to extended exposure to hypobaric hypoxia itself (10-91 days), endurance training in hypobaric hypoxic conditions leads to a marked early increase in ventricular and atrial BNP mRNA levels.[21] As for the physiologic effects that can be produced by elevated plasma BNP levels, Klinger *et al*., found that in rats, BNP infusion attenuated the development of hypoxic pulmonary hypertension.[22] The authors suggested that this finding supports the hypothesis that endogenous BNP plays a role in modulating the pulmonary hypertensive responses seen in chronic hypoxia. With these findings in mind, we hypothesized that under hypobaric hypoxic conditions, endogenous BNP might serve to compensate the rise in IOP resulting from exposure to these conditions, because we were anticipating a significant change in BNP due to hypoxia. In our study, plasma BNP levels did not change after short-term hypobaric hypoxic exposure, because of this, there has not been any compensation against the IOP increase which probably has been caused by some other systemic factors. One possible reason for the lack of significant change in BNP levels might be the relatively short period of exposure to the hypobaric hypoxic conditions. As the hypobaric hypoxic conditions were reversed, IOP decreased to normal levels. When considering other factors that could have acted in the IOP increase, one should take into account the physiological responses to acute hypoxia. The hypoxia induces both general and regional changes in the cardiovascular and respiratory systems. The heart rate increases as the partial oxygen pressure decreases, and it is doubled even below 30000 ft. There is also a proportional increase in cardiac output. The systolic pressure and the pulse pressure are raised too. By the increasing altitude, respiratory rate shows an increase and decrease at mild to moderate hypoxia, but minute volume increases steadily. The hyperpnea, tachypnea, increased heart rate and increased cardiac output are mainly the results of carotid and aortic chemoreceptor stimulation.[24] In addition, it is known that plasma cortisol levels cause the diurnal variation in IOP[27] and exposure to hypobaric hypoxia influences cortisol levels.[28] These systemic changes might be possibly causing the increase in IOP. Probably, as these factors are restored after the end of hypoxic exposure, IOP returns to normal levels.

To the best of our knowledge, this is the first study to evaluate the relation between plasma BNP and changes in IOP due to hypobaric hypoxic exposure. Therefore, it is not currently possible to compare our data directly with those of other studies.

## Conclusion

Short-term hypobaric hypoxic exposure did not cause an increase in plasma BNP levels, probably the increased IOP levels are compensated by some local or systemic factors other than BNP levels. IOP decreased to normal levels as the ambient pressure returned to normal conditions. Further studies are needed to determine the exact factors and compensation mechanisms causing the IOP increase in hypobaric hypoxic conditions.
